# LncRNA AC100826.1 regulated PLCB1 to promote progression in non‐small cell lung cancer

**DOI:** 10.1111/1759-7714.15323

**Published:** 2024-05-22

**Authors:** Shenhui Dai, Qiao Wang, Yin Lyu, Zhipeng Chen, Xiucheng Liu, Guoqing Zhao, Hao Zhang

**Affiliations:** ^1^ Thoracic Surgery Laboratory, Xuzhou Medical University Xuzhou China; ^2^ Department of Thoracic Surgery Affiliated Hospital of Xuzhou Medical University Xuzhou China

**Keywords:** Lnc AC100826.1, migration, NSCLC, PLCB1

## Abstract

**Background:**

Lung cancer is the most common malignant tumor. In the present study, we identified a long non‐coding RNA (lncRNA) AC100826.1 (simplify to Lnc1), which was highly expressed in non‐small cell lung cancer (NSCLC) tissues compared with the paracancerous tissues. We also observed the critical role of Lnc1 in regulating the metastasis ability of NSCLC cells.

**Methods:**

RNA sequencing was performed to detect differential expression levels of lncRNAs in NSCLC tissues and its paracancerous tissues. Effects of Lnc1 on cell proliferation, invasion, and migration were determined by CCK‐8, transwell and scratch assays. The xenograft experiment confirmed the effect of Lnc1 on NSCLC cells proliferation and migration abilities in vivo. RT‐qPCR and western blots were performed to determine the expression levels of mRNAs and proteins.

**Results:**

The expression level of Lnc1 was related to multiple pathological results, knockdown of Lnc1 can inhibit the proliferation and metastasis abilities of NSCLC cells. silencing phospholipase C, β1(PLCB1) can reverse the promoting effects of overexpression Lnc1 on NSCLC cells proliferation and migration abilities. In addition, the Rap1 signaling pathway was implicated in the regulation of Lnc1 in NSCLC metastasis.

**Conclusion:**

Our results suggest that Lnc1 regulated the metastatic ability of NSCLC cells through targeting the PLCB1/Rap1 signal pathway.

## Graphical abstract

Lnc1 mediates the metastasis of NSCLC through regulating the PLCB1/Rap1 signaling pathway, while a inhibitor (IN‐3) of the Rap1 signaling pathway, can reverse the changes in cadherin caused by overexpression of Lnc1.

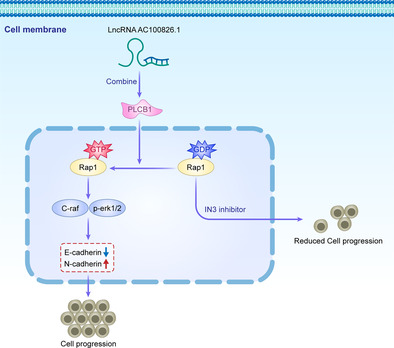

## INTRODUCTION

Lung cancer is currently the most fatal malignant tumor, compared with other tumors and has a significantly higher mortality rate.^1^, ^2^, ^3^ Among all types of lung cancer, non‐small cell lung cancer (NSCLC) accounts for a high proportion.^4^, ^5^ At present, chemotherapy and radiotherapy are still the mainstream treatment methods for advanced lung cancer patients,^6^ and the side effects of chemotherapy bring huge physical burden to patients, such as vomiting, loss of hair and severe neuropathy. In addition, the formation of chemotherapy drug resistance is the main obstacle to increasing the overall survival time of lung patients.

Several studies have reported that long noncoding RNAs (lncRNAs) are closely related to the occurrence of many malignant tumors, such as osteosarcoma,^7^ breast cancer,^8^ ovarian cancer^9^ and NSCLC.^10^ LncRNA 408 promotes invasion and metastasis of breast cancer cell by regulating LIMK1.^11^ LncRNA CPLC promotes the progression of colorectal cancer via regulating ZBTB34 by competitively binding miR‐4319.^12^ Therefore, the study of the presence, functional mechanism and clinical significance of lncRNAs in NSCLC, which provide new biomarkers and therapeutic targets for the treatment and prognosis of NSCLC, is pivotal in the pathogenesis of NSCLC.

We found that LncRNA AC100826.1 (ENST00000558010.1, simplify to Lnc1) was expressed in a high level in NSCLC tissues compared with the paracancerous tissues by RNA sequencing (RNAseq). Follow‐up prediction phospholipase C, β1(PLCB1) is a potential regulatory target of Lnc1 by LncTarget data, and it catalyzes the formation of inositol 1,4,5‐trisphosphate and diacylglycerol.^13^, ^14^, ^15^ Thus, PLCB1 is related to the function of many extracellular signals in the intracellular transduction.^15^, ^16^, ^17^ In addition, we identified that PLCB1 was highly expressed in NSCLC tissues and relative cell lines. The results showed that PLCB1 promoted the proliferation and migration of the NSCLC cells. We also found that the Rap1 signaling pathway may participate regulation of Lnc1 in the NSCLC through Kyoto Encyclopedia of Genes and Genomes (KEGG) pathway enrichment analysis. Rap1 proteins are archetypes of a superfamily of small GTPases. Previous research has shown that the physiological functions mediated by Rap1 signaling plays different regulatory roles in tumorigenic processes,^18^, ^19^, ^20^ especially in the proliferation of tumor cells and metastatic deterioration process of tumor cells.^21^, ^22^, ^23^ For example, Mex3a promotes oncogenesis through the Rap1 signaling pathway in colorectal cancer and is inhibited by miR‐6887‐3p.^24^ Loss of TTC17 promotes breast cancer metastasis through the Rap1 signal pathway.^25^ Therefore, Rap1 plays a very important part in tumor cell genesis and metastasis. In summary, in the present study, we further clarified the function and mechanism of Lnc1 in the occurrence and development of NSCLC, and explored its clinical significance, providing new ideas for the pathogenesis of NSCLC.

## METHODS

### Pathological specimen

The tumor and paracancerous tissues were collected from patients diagnosed with NSCLC in the Affiliated Hospital of Xuzhou Medical University between 2021 and 2022. All samples were collected during the operation and immediately stored in liquid nitrogen for follow‐up experiments. The clinical trials were approved by the Ethics Committee of the Affiliated Hospital of Xuzhou Medical University, and the detailed information of the patients are summarized in Table 1.

**TABLE 1:** Primer sequences for reverse transcription‐quantitative polymerase chain reaction.

Gene		Primer sequences (strand)
Lnc1	Forward	5′‐ATGCTGAACAGCGGAACACA‐3′
	Reverse	5′‐TTCTGAGCTGATGCCCACTC‐3′
PLCB1	Forward	5′‐AAGGCCGTGTGCGTGCTGAA‐3′
	Reverse	5′‐GGCCCACCGTGTTTTCTGGA‐3′
ANCY7	Forward	5′‐GCGGGGCAGGTACTACTTA‐3′
	Reverse	5′‐CTCTTCGTTCTTGGCGTTCT‐3′
NGF	Forward	5′‐GTGCGGTGCGTGCCCTACTT‐3′
	Reverse	5′‐GCTCTTCCAGTGCCTTCGT‐3′
TLN2	Forward	5′‐CTCGCTTCGGCAGCACA‐3′
	Reverse	5′‐AACGCTTCACGAATTTGCGT‐3′
Z99127	Forward	5′‐ATTTGGACATCAGGAGAACTGT‐3′
	Reverse	5′‐CTTCAGGTAGGCGAAGACA‐3′
AC084782	Forward	5′‐GCATTGTGTGCCATGGTGAG‐3′
	Reverse	5′‐CCGAGTGTGAGCATCATGGC‐3′
AL049830	Forward	5′‐CCGATGACTCATCTATCTGG‐3′
	Reverse	5′‐ATTAGCCGATCGATCGGGCA‐3′

### Cell culture and vector transfection

A549, H1299, H292, H23 and BEAS‐2B cell lines were purchased from the Chinese Academy of Sciences Shanghai Cell Bank. All cells were cultured in medium containing 5% fetal bovine serum (FBS) in an incubator at 37°C, 5% CO_2_ environment. The sequence of Lnc1 was cloned into an adenoviral vector to construct Lnc1 overexpression and knockdown vectors (Jima). Small interfering RNA (SiRNA) specifically targeting Lnc1 and PLCB1 was purchased from Sangon Biotechnology. Corresponding SiRNA transfection of A549 and H1299 cell lines was performed according to the manufacturer's instructions. The relative expression of Lnc1 knockdown and overexpression were identified by reverse transcription‐quantitative polymerase chain reaction (RT‐qPCR) (Figure S1D).

### RT‐qPCR

Total RNA was extracted using a TRIzol kit (Invitrogen). After total RNA was reverse transcribed to Cdna by PrimeScript RT reagent kit (Promega), the RNA concentration and purity were determined by Nanodrop. The primer sequences of the target genes are listed in Table 1, Lnc1 shRNA sequences are listed in Table 2.

**TABLE 2:** Lnc1 shRNA sequences.

	Sense	Anti‐sense
shCtrl	CCGGCCTAAGGTTAAGTCGCCCTCTCGAGAGCGAGG	AATTCAAAAACCTAAGGTTAAGTCGCCCTCTCGAGAGCC
shLnc1#1	CCGGGCAGCTCAGGTGTATGTAAGGCTCGAGCCTTACATACACCTGAGCTGCTTTTTG	AATTCAAAAAGCAGCTCAGGTGTATGTAAGGCTCGAGCCTTACATACACCTGAGCTGC
shLnc1#2	CCGGGCACCAAGAGAGCCCTAAAGACTCGAGTCTTTAGGGCTCTCTTGGTGCTTTTTG	AATTCAAAAAGCACCAAGAGAGCCCTAAAGACTCGAGTCTTTAGGGCTCTCTTGGTGC

### Western blots

Radioimmunoprecipitation assay (RIPA) lysis buffer (Beyotime) was used to extract total proteins. Sodium sulfate polyacrylamide gel electrophoresis (SDS‐PAGE) was used to separate target proteins. Then, nitrocellulose filter membranes were incubated with primary antibodies, including anti‐E‐cadherin (20874‐1‐ap, Proteintech), anti‐N‐cadherin (22018‐1‐ap, Proteintech), anti‐PLCB1 (26551‐1‐ap, Proteintech), anti‐ERK1/2 (11257‐1‐ap, Proteintech), antiRap1 (10840‐1‐ap, Proteintech), anti‐c‐raf (26863‐1‐ap, Proteintech) and anti‐α‐tublin (11224‐1‐ap, Proteintech) and anti‐GAPDH (10494‐1‐ap, Proteintech). Secondary antibodies, including HRP goat anti‐mouse IgG and HRP goat anti‐rabbit IgG, were used for detection. The protein bands were observed using an enhanced chemiluminescence (ECL) system (Bio‐Rad). Finally, all protein bands were quantified using Image J software.

### Measurement of cell proliferation ability

A cell counting kit‐8 (CCK‐8) assay was performed to detect cell proliferation to measure the absorbance of the NSCLC cell lines by using a spectrophotometer at OD 450. Concrete steps: NSCLC cells were diluted to 4000 cells/200 μL and inoculated into a 96 well plate. NSCLC cells were incubated at 37°C for 0, 24, 48, 72 h. A 5‐ethynyl‐2‐deoxyuridine (EDU) assay was used to detect cell proliferation and the steps performed according to the reagent manufacturer's instructions (Ribobio).

### Measurement of cell migration and invasion abilities

Corresponding shRNA gene was transfected into A549 and H1299 cells. For the migration assays, 5 × 10^4^ cells were placed in the upper chamber in 200 μL medium without FBS. For the invasion assay, 5 × 10^4^ cells were placed in the upper chamber with solidified matrix glue, and 800 μL of culture medium containing 25% FBS were placed into the lower chamber. After incubation for 48 h, cells remaining on the upper membrane were removed by cotton swab, and the lower membrane was stained with 1% crystal violet (Beyotime) at room temperature for 30 min. 2 or 3 random fields per chamber were counted by fluorescent microscope (Olympus). For the would healing assay, 5 × 10^5^ cells were placed into six‐well plates and cultured overnight. When the cell density reached 90%, a sterile 1 mL disposable pipette tip was used to produce uniform scratches. After the incubation of cells for 24 and 48 h in FBS medium containing 1%, a microscope was used to obtain images of the plate (Olympus).

### Construction of the mice tumor model

This experiment was in accordance with the regulations of Committee the Xuzhou Medical University Experimental Animal Use and Care. The male BALB/c nude mice were 5 weeks old, and the mice at the SPF level for 1 week. Subcutaneous (*n* = 20, 2 × 10^6^ cell/mouse) or tail vein (*n* = 6, 2 × 10^6^ cell/mouse) were injected with A549‐shControl cells and A549‐shLnc1 cells, respectively. The mice were killed by CO_2_ after 4–5 weeks. The tumor and lung were removed and fixed in 4% formalin solution then observed using hematoxylin and eosin (HE) staining and immunohistochemistry.

### Immunohistochemistry

Paraffin sections were prepared, and the slide was then placed in a graded series of xylene for dewaxing. It was then placed in an alcohol solution of different concentrations (100%, 95%, 80%, 75% ethanol solution, respectively). Next, it was incubated with citric acid tissue antigen repair solution and sealing solution at 37°C for 30 min. PBS was used to wash it three times for 10 min each time, and an antibody (E‐cadherin, N‐cadherin, Ki67) added, before being stored at 4°C overnight. The slide was then removed and washed with PBS three times. Then, the second antibody was added and incubated at 37°C for 30 min. The slides were removed from the incubator, washed three times with PBS, and then DAB liquid configured. The neutral resin was dropped onto the tissue section, and the slide placed in a fume hood to dry.

### RNA pulldown and RIP

A magnetic RNA‐protein pulldown kit (Thermo Scientific) was used to pull down RNA according to the manufacturer's instructions. The target band was first identified by Coomassie brilliant blue staining and then quantitated by western blot. RNA immunoprecipitation (RIP) was carried out according to the manufacturer's protocol using RIP RNA binding protein immunoprecipitation kit (Thermo Scientific).

### Statistical analysis

GraphPad Prism 8.0 software and SPSS 26.0 software were used for data processing. The data of the two groups were compared by the *t* test. The data of three groups and more groups were compared by one‐way analysis of variance (ANOVA). The enumeration data was compared by chi‐square test. *p* < 0.05 was considered statistically significant. Survival analysis for PLCB1 in NSCLC was performed using the Kaplan–Meier Plotter online database (http://kmplot.com/analysis/index.php?p=background).

## RESULTS

### Lnc1 expression is high in NSCLC tissues and relative cell lines

**FIGURE 1:**
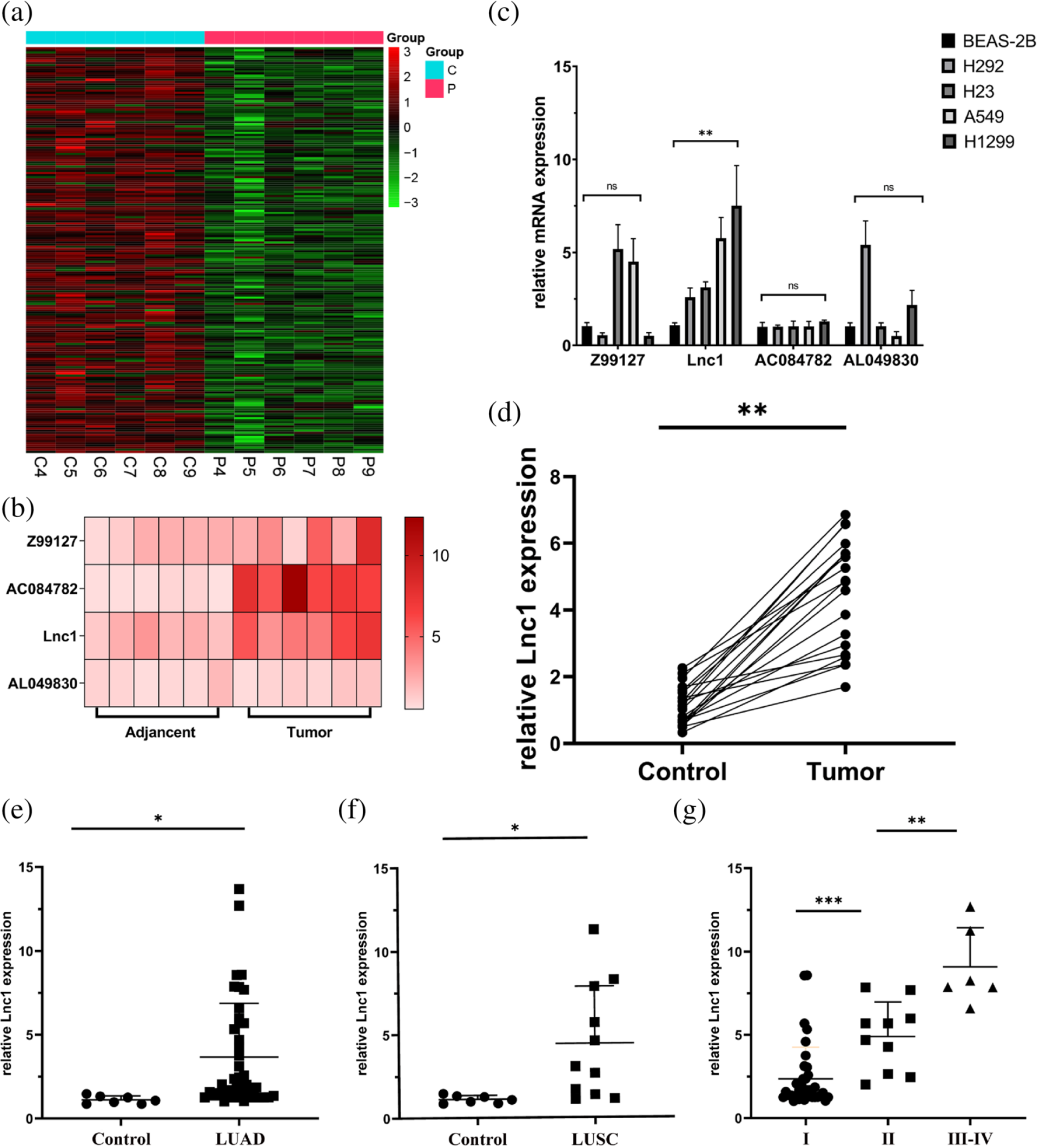
Lnc1 expression was high in non‐small cell lung cancer (NSCLC) tissues and relative cell lines. (a, b) RNAseq was used to screen LncRNAs in tissues and paracancerous tissues of NSCLC patients. (c) The expression levels of LncRNAs in different subtypes of human NSCLC cell lines by reverse transcription‐quantitative polymerase chain reaction (RT‐qRCR). (d) Expression levels of Lnc1 in 20 clinical patients by RT‐qPCR. (e, f) Expression levels of Lnc1 were verified according to pathological type. LUAD, lung adenocarcinoma; LUSC, lung squamous carcinoma. (g) Expression levels of Lnc1 were verified according to TNM stage (ns, no statistical significance, *p < 0.05, **p < 0.01).

By RNAseq analysis of NSCLC tissues and its paracancerous tissues, we selected the top four LncRNAs with the most significant differential expression. The LncRNAs were Z99127, Lnc1, AC084782 and AL049830 (Figure 1a,b). Next, four different subtypes of human NSCLC (H292, H23, A549 and H1299) were examined to determine whether Z99127, Lnc1, AC084782 and AL049830 levels expression were similarly associated as observed in RNAseq analysis. The results suggested that only Lnc1 was stably high expressed in four different subtypes of human NSCLC (Figure 1c). To further characterize Lnc1 in human NSCLC, the expression level of Lnc1 in NSCLC tissues higher than the adjacent tissues of 20 patient samples by RT‐qPCR (Figure 1d). The above research indicated that Lnc1 was highly expressed in NSCLC tissues and related cell lines. Due to the highest expression level of Lnc1 in A549 and H1299 cells, we chose to knockdown Lnc1 in A549 and H1299 cells for further experiments.

The expression of Lnc1 was detected in 53 NSCLC patients to evaluate the clinical significance, and the Lnc1 expression level was significantly associated with the pathological characteristics (Table 3). The median expression level of the Lnc1 was considered as a key value, and the samples were divided into low expression groups (26 samples) and high expression groups (27 samples) as indicated in Table 3. High expression of Lnc1 were significantly related with lymph node metastasis (*p* = 0.025), tumor size (*p* = 0.034), degree of differentiation (*p* = 0.001) and distant metastasis (*p* = 0.028). However, there was no obvious association between Lnc1 expression and other features, including gender (*p* = 0.126) and age (*p* = 0.678). We also found that Lnc1 was highly expressed in lung adenocarcinoma (LUAD) and lung squamous carcinoma (LUSC) (Figure 1e,f). In addition, tumor node metastasis (TNM) classification was also related with the expression of Lnc1 and increased with advanced TNM stage (Figure 1g). In general, Lnc1 was strongly expressed in NSCLC tissues, and the four subtypes of NSCLC cell lines also with high Lnc1 expression, thus, supporting the findings at mRNA level.

**TABLE 3:** The relationship between Lnc1 expression and pathological characteristics of non‐small cell lung cancer.

Features	Number	Lnc1 expression level		*X* ^2^	*p*‐value
		Low	High		
Gender					
Male	24	9	15	2.344	0.126
Female	29	17	12		
Age (years)					
≥60	26	12	14	0.172	0.678
<60	27	14	13		
Tissue differentiation					
Well	11	10	1	14.348	0.001
Moderate	27	13	14		
Poor	15	3	12		
Pathological type					
LUAD	41	22	19	1.535	0.215
LUSC	12	4	8		
Tumor size (cm)					
≤3	34	21	13	6.771	0.034
3–5	12	4	8		
>5	7	1	6		
Node metastasis					
Yes	8	1	7	5.038	0.025
No	45	25	20		
Distant metastasis					
Yes	5	0	5	‐	0.028
No	48	26	22		

Abbreviations: LUAD, lung adenocarcinoma; LUSC, lung squamous carcinoma.

### Lnc1 promotes the proliferation, invasion and migration of NSCLC cells

**FIGURE 2:**
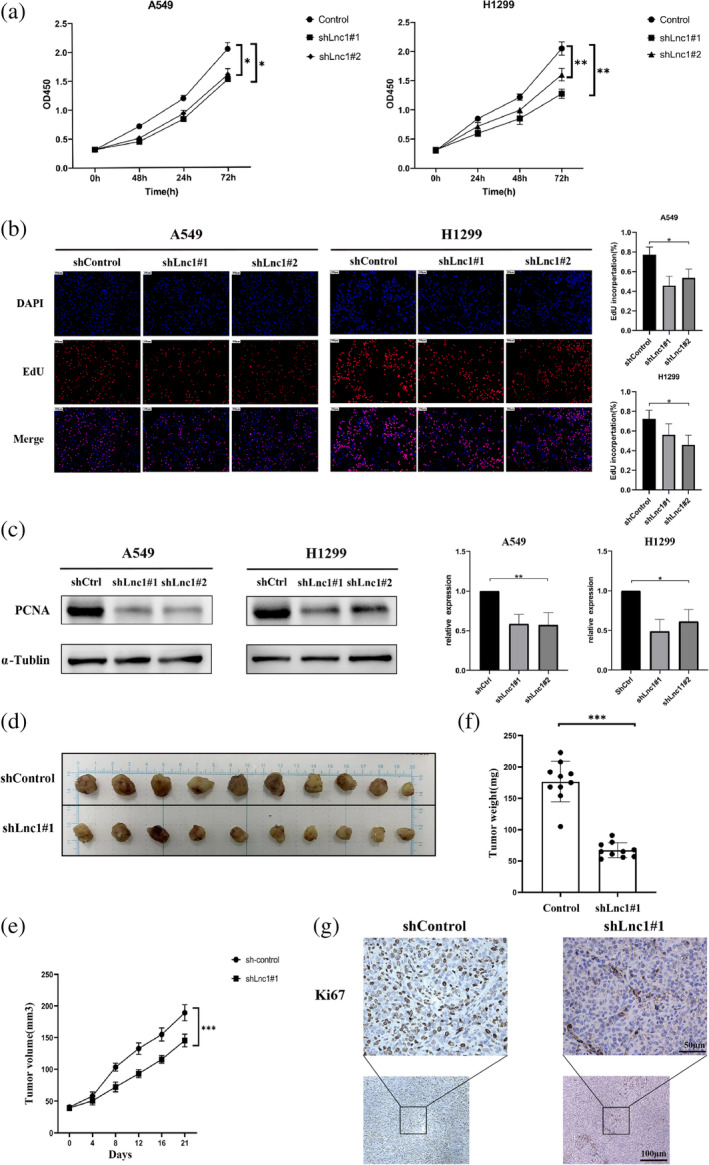
Lnc1 promotes the proliferation of non‐small cell lung cancer (NSCLC) cells. (a, b) Cell counting kit‐8 (CCK‐8) and 5‐ethynyl‐2‐deoxyuridine (EDU) assays were performed to evaluate the effect of Lnc1 on the proliferation of A549 cells and H1299 cells. (c) Western blots were used to analyze the expression level of proliferation‐related protein PCNA in A549 cells and H1299 cells. (d–f) Subcutaneous tumorigenesis model in nude mice showed that knockdown of Lnc1 decreased tumor growth, including tumor weights and volumes. (g) The xenografts were subjected to immunohistochemical (IHC) staining with Ki‐67 (ns, statistical significance, *p < 0.05, **p < 0.01).

To investigate the effects of Lnc1 in different subtypes of human NSCLC cell lines, we chose to knockdown Lnc1 in A549 and H1299 cells. The results showed that downregulation of Lnc1 could inhibit the proliferation of human NSCLC cell lines via CCK‐8 and EDU assays (Figure 2a,b). Western blot results also showed that the expression of proliferating cell nuclear antigen (PCNA), a cell proliferation biomarker, was downregulated in A549 and H1299 cells after knockdown of Lnc1 (Figure 2c). To further verify that Lnc1 promoted the proliferation of NSCLC cells in vivo, we injected Lnc1 knockdown cells into the subcutaneous tissue of the nude mice. The results were as expected: the volumes and weights of the tumors were significantly decreased compared with the control groups (Figure 2d–f). The Ki‐67 index was lower in the shControl group than the shLnc1#1 group (Figure 2g). These results suggested that inhibiting Lnc1 expression can significantly suppress the proliferation ability of NSCLC cells both in vitro and in vivo.

**FIGURE 3:**
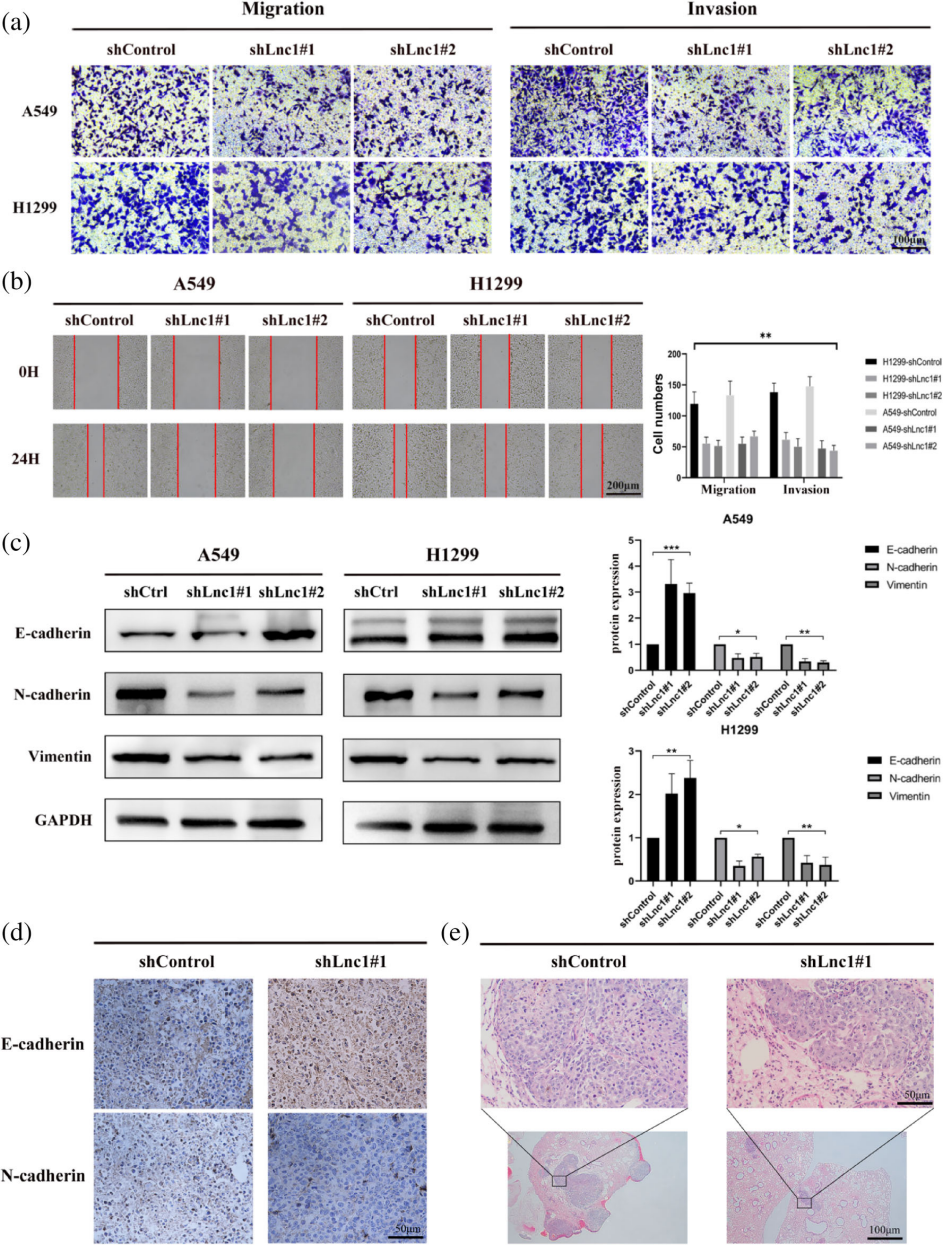
Lnc1 promotes the migration and invasion of non‐small cell lung cancer (NSCLC) cells. (a) Transwell assay was used to measure the migration and invasion of A549 cells and H1229 cells. (b) The migration ability of A549 cells and H1229 cells was tested by cell scratch assay. (c) Western blots were used to detect the expression level of epithelial mesenchymal transition (EMT)‐related proteins in A549 cells and H1299 cells, including E‐cadherin, N‐cadherin and vimentin. (d) Immunohistochemistry was used to detect the changes of tumor EMT‐related proteins in the xenografts (E‐cadherin and N‐cadherin). (e) Hematoxylin and eosin staining showed lung tumor metastasis in mice (ns, no statistical significance, *p < 0.05, **p < 0.01).

Next, we conducted experiments related to tumor metastasis. The downregulation of Lnc1 significantly reduced migration and invasion ability of different subtypes of human NSCLC cell lines compared with the shControl group via transwell and cell scratch assays (Figures 3a,b, S1F). From these results, we assumed that Lnc1 might be involved in the metastatic mechanisms of NSCLC. As is well known, tumor cell metastasis is related to epithelial mesenchymal transition (EMT). Thus, we detected the EMT related proteins (E‐cadherin, N‐cadherin and vimentin) by western blots. The results showed that E‐cadherin was increased after Lnc1 inhibition in A549 and H1299 cells, and N‐cadherin and vimentin decreased after Lnc1 inhibition in A549 and H1299 cells (Figure 3c). To determine whether Lnc1 can promote tumor metastasis in vivo, we used adenoviral vector to transfect shLnc1#1 into A549 cells to knockdown the expression of the shLnc1. The cells were intravenously injected into BALB/c mice. The immunohistochemistry (IHC) of mice tumors also showed that E‐cadherin increased expression, and N‐cadherin decreased expression in the shLnc1#1 group (Figure 3d). Hematoxylin and eosin (HE) staining indicated that the number and size of lung tumors produced in the shLnc1#1 group were significantly weaker compared with the shControl group (Figures 3e, S1E). In summary, we concluded that Lnc1 can promote tumor proliferation and metastasis in vitro and in vivo.

### Lnc1 promotes tumor proliferation and migration mediated by PLCB1

**FIGURE 4:**
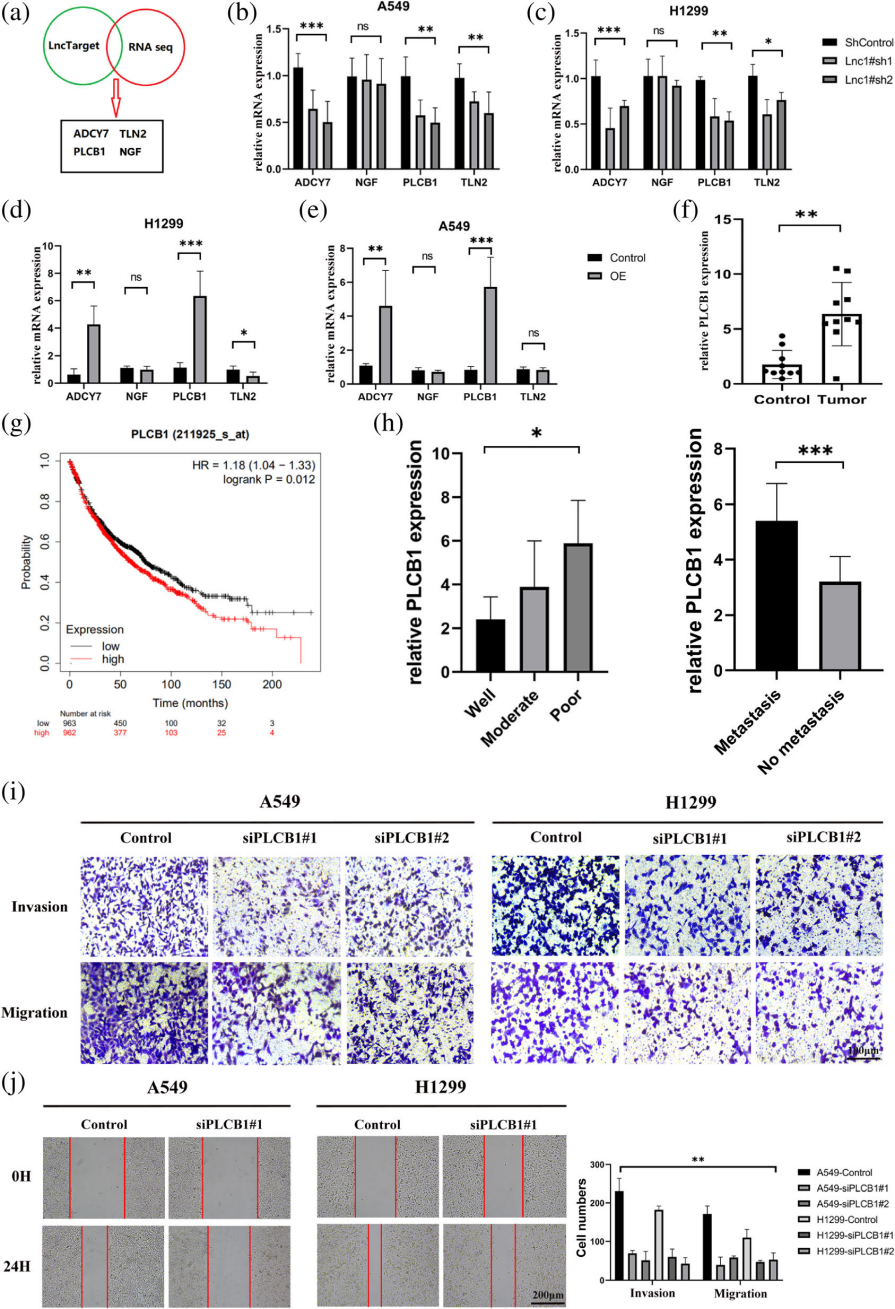
Lnc1 promotes non‐small cell lung cancer (NSCLC) cell proliferation and metastasis mediated by PLCB1. (a) Identifying potential target genes of the downstream of Lnc1 (Green:LncTarget data; Red: RNAseq). (b–e) Using reverse transcription‐quantitative polymerase chain reaction (RT‐qPCR) to quantifiability detection the level of potential target genes (ADCY7, NGF, PLCB1 and TLN2) after knockdown or overexpression of Lnc1 in A549 cells and H1299 cells. (f) Quantifiability detection of the mRNA expression level of PLCB1 in 10 pairs of clinical samples. (g) The prognostic value of PLCB1 in NSCLC examined by Kaplan–Meier Plotter. (h) The expression level of PLCB1 in patients with different degrees of differentiation and metastasis. (i, j) Transwell and scratch assays proved that PLCB1 can promote tumor cell migration (ns, no statistical significance, *p < 0.05, **p < 0.01, ***p < 0.001).

To research the detailed molecular mechanism of Lnc1 promoting NSCLC progression, we predicted the directly interacting potential target genes with Lnc1 by RNAseq and LncTarget prediction data (http://www.cuilab.cn/lnctar). Four candidate target genes (NGF, PLCB1, ACDY7 and TLN2) were screened (Figure 4a). To verify the specificity of the above candidate genes with Lnc1, the expression levels of the candidate target genes and its correlation plots with Lnc1 in NSCLC cells were examined by RT‐qPCR (Figure 4b–e). We found that only ACDY7 and PLCB1 were positively associated with the Lnc1 expression. Next, we initially verified the relationship between the two candidate target genes (ACDY7 and PLCB1) by interfering its expression. We found that inhibited ADCY7 had no effect on cell proliferation and migration abilities (Figure S1A,B). Inhibited PLCB1 could downregulate cell proliferation and migration abilities.

**FIGURE 5:**
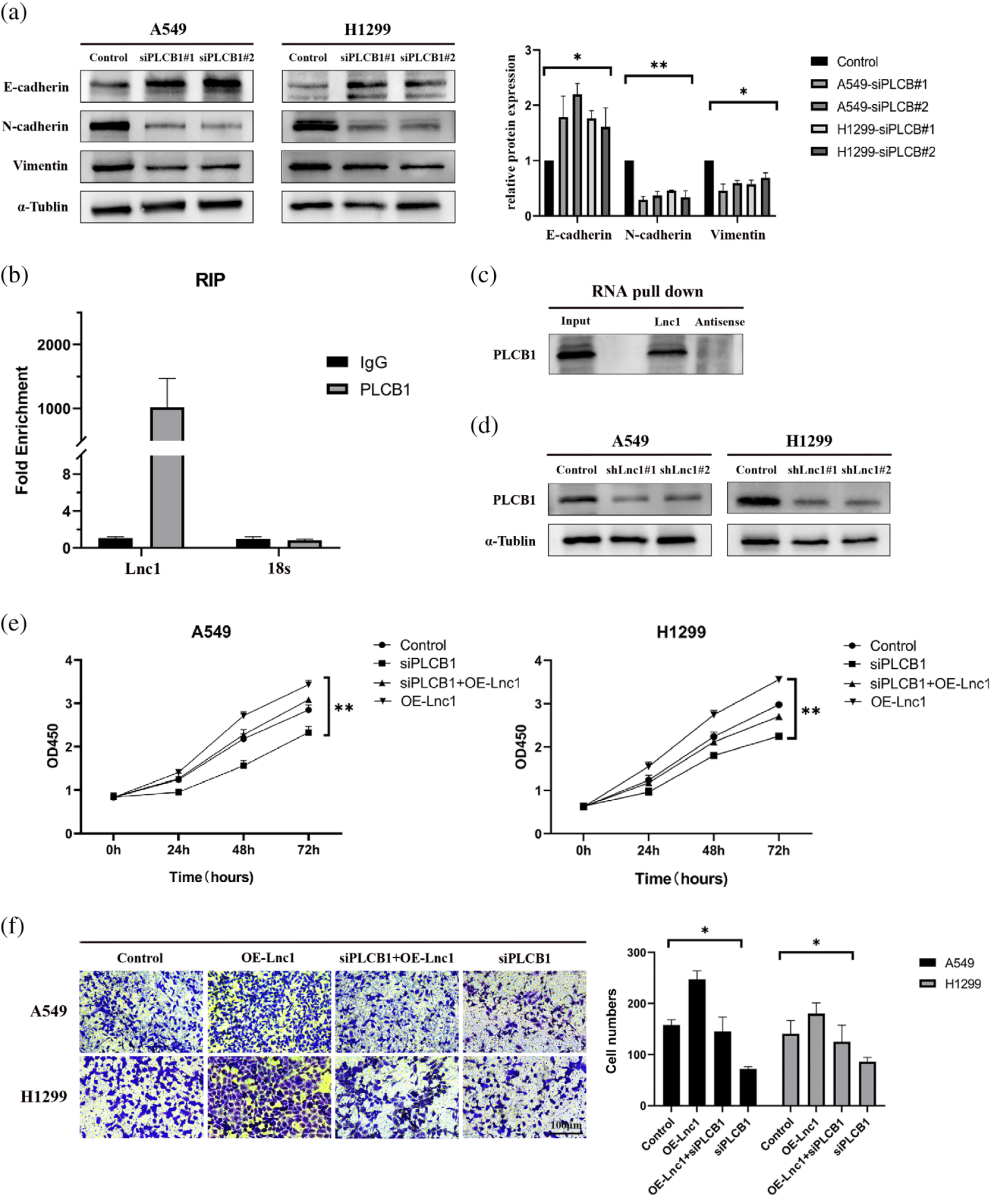
Lnc1 promotes non‐small cell lung cancer (NSCLC) cell proliferation and metastasis mediated by PLCB1. (a) Western blots were used to analyze the expression level of epithelial mesenchymal transition (EMT)‐related proteins in A549 cells and H1299 cells. (b, c) RNA pulldown and RNA immunoprecipitation (RIP) experiments proved the interaction between PLCB1 and Lnc1. (d) Western blots were used to analyze the protein expression level of PLCB1 after knockdown Lnc1 in A549 cell and H1299 cell. (e, f) Cell counting kit‐8 (CCK‐8) and transwell assays were used to evaluate the interaction PLCB1 and Lnc1 on A549 cell and H1299 cell.(ns, no statistical significance, *p < 0.05, **p < 0.01).

Moreover, we selected 10 pairs of patients, pathologically diagnosed as NSCLC and benign tumor in clinical, to analyze the PLCB1 expression by RT‐qPCR. The results showed that PLCB1 expressed at a high level in the tumor tissues (Figure 4f). The Kaplan–Meier plotter showed that high expression of PLCB1 in NSCLC would lead to poor prognosis (Figure 4g). We found that PLCB1 was correlated with the degree of tumor differentiation and tumor metastasis by analyzing the expression level of PLCB1 in NSCLC patients (Figure 4h). The above factors showed that the expression level of PLCB1 remained an important prognostic factor for the survival of NSCLC patients. Knockdown of PLCB1 in A549 cells and H1299 cells to further research the biological function of PLCB1 in NSCLC cells was performed. Transwell and scratch assays showed that downregulation of PLCB1 significantly inhibited migration and invasion of A549 cells and H1299 cells compared with the control group (Figures 4i,j, S1g). We found increased expression in E‐cadherin after inhibition of PLCB1 expression, but decreased expression in N‐cadherin and vimentin (Figure 5a). The results suggest that the downregulation of PLCB1 can inhibit the migration of A549 cell and H1299 cells.

Although we found that PLCB1 promoted the proliferation and metastasis of NSCLC related cell lines cell lines, its specific mechanism remained unclear. We used the online database to predict the interaction between Lnc1 and PLCB1 (http://pridb.gdcb.iastate.edu/RPISeq/). The result indicated that Lnc1 and PLCB1 were more likely to interact. Significant enrichment of Lnc1 in RNA‐protein complexes precipitated with antibody against PLCB1 as compared with the IgG control (Figure 5b). We further performed RNA pulldown assay with the A549 cells extracts and observed that there were several proteins were pulleddown by Lnc1 (Figure 5c). Among them, the protein bands with molecular weight between 100 KDa‐150 KDa were the most obvious, and PLCB1 was also in the range (Figure S1c). Next, PLCB1 was proved in A549 cells through western blots. We found that downregulation of Lnc1 effectively reduced PLCB1 protein levels in A549 cells and H1299 cells (Figure 5d). Above all, our results suggested that Lnc1 directly binds PLCB1 and regulated the expression of PLCB1 in the upstream level. Next, to investigate whether Lnc1 regulated the proliferate and metastatic ability of tumor cells in a PLCB1‐mediated manner. The results showed that inhibition of PLCB1 can reverse the enhanced proliferation and migration of Lnc1 overexpression in A549 cells and H1299 cells by CCK‐8 and transwell asssys (Figure 5e,f). In conclusion, Lnc1 promoted tumor proliferation and migration mediated by PLCB1.

### Lnc1 activates Rap1 signaling pathway by targeting PLCB1

**FIGURE 6:**
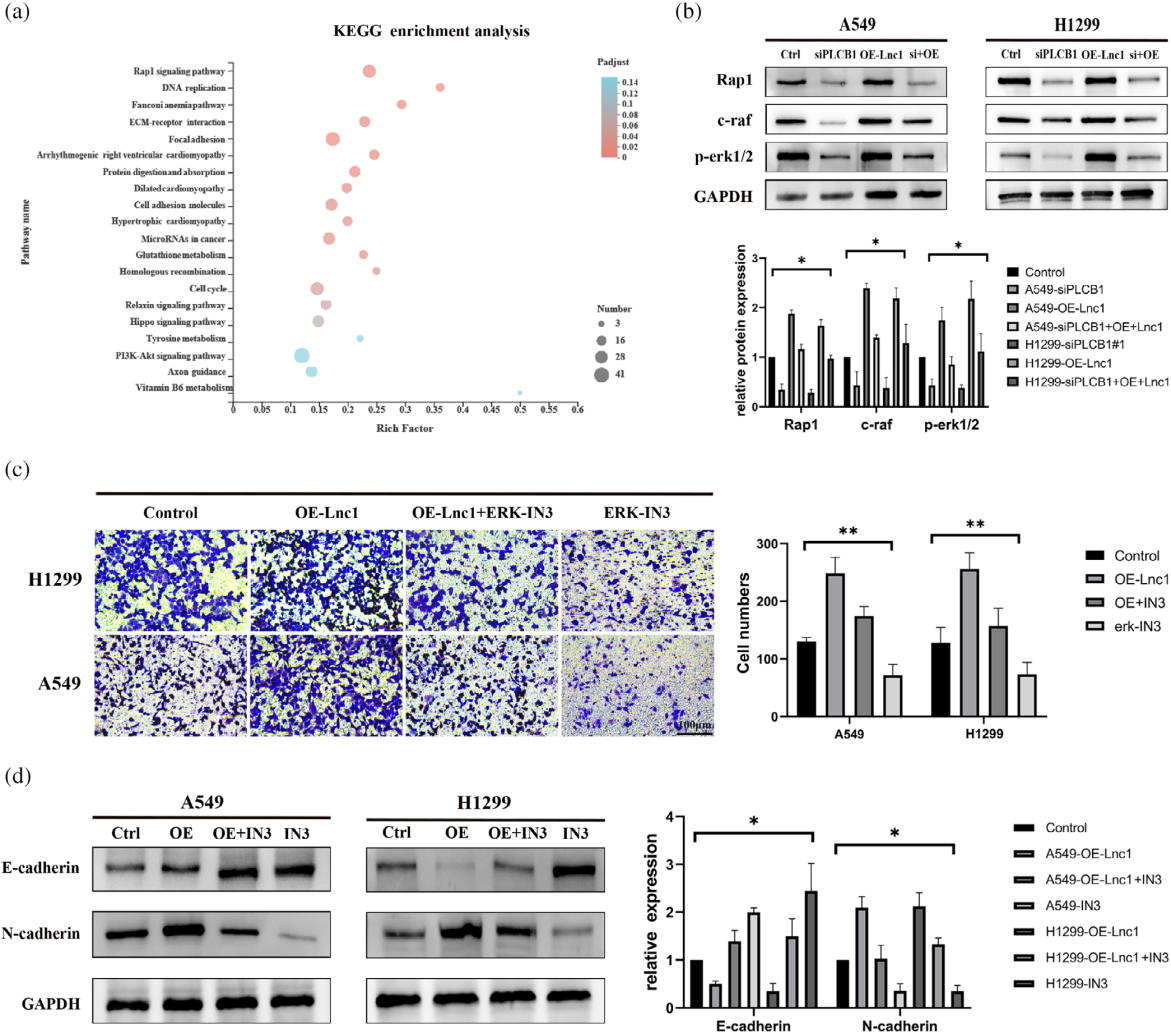
Lnc1 activates Rap1 signaling pathway by targeting PLCB1. (a) Kyoto Encyclopedia of Genes and Genomes (KEGG) pathway enrichment analysis was used to detect the signal regulation in shControl‐A549 group and shLnc1#1‐A549 group. (b) Western blots were used to analyze the expression level of Rap1 signal pathway marker protein in A549 cells and H1299 cells. (c) Transwell assay was used to measure the migration of A549 cells and H1229 cells after treatment with the Rap1 signaling pathway inhibitor (ERK‐IN3). (d) Western blots were used to evaluate the effect of the Rap1 signaling pathway inhibitor (ERK‐IN3) on epithelial mesenchymal transition (EMT) related proteins. (ns, no statistical significance, *p < 0.05, **p < 0.01).

To identify the downstream signal pathway of Lnc1, we analyzed the changes in signal pathways and functions through KEGG analysis in the shControl group and shLnc1#1 groups. The analysis found that the Rap1 signal pathway participates in the regulation of Lnc1/PLCB1 (Figure 6a). It has previously been reported that Rap1 mediates adhesion junctions and tight junctions of epithelial cells and endothelial cells, and plays a major role in the protection of the endothelial barrier.^20^ In addition, Rap1 acts as a key factor that affect the biological properties of adhesion and invasion in a variety of malignant cells.^21^, ^22^ The main signal molecules of Rap1 pathway were rap1, erk1/2 and c‐raf. Western blots showed that the expression of major protein (Rap1, erk1/2 and c‐raf) were decreased in A549 cells and H1299 cells after silencing PLCB1. In addition, Lnc1 overexpression significantly promoted the expression of rap1, erk1/2 and c‐raf. Vice versa, silencing PLCB1 reversed the increased expression of rap1, erk1/2 and c‐raf expression in A549 cells and H1299 cells caused by Lnc1 overexpression (Figure 6b). We used ERK‐IN‐3, which was an inhibitor of erk1/2, to interfere the the Rap1 signaling pathway. We found that inhibition of the Rap1 signaling pathway diminished the metastasis ability of A549 cells and H1299 cells, and the ERK‐IN‐3 also can reverse the enhanced metastasis ability of Lnc1 overexpression (Figure 6c). Obviously, the ERK‐IN‐3 can also increase the expression of E‐cadherin and decrease the expression of N‐cadherin. In addition, ERK‐IN3 can also reverse the changes of cadherin level caused by the overexpression of Lnc1 and the appearance was consistent with the trend after silencing PLCB1 (Figure 6d). The above results indicated that Lnc1 regulated the metastatic ability of NSCLC cells through targeting PLCB1/Rap1 signaling pathway.

## DISCUSSION

NSCLC has a detrimental impact on human health.^26^ However, due to the complexity of the pathogenesis of NSCLC, to date, there are no effective drugs to treat NSCLC patients. Therefore, revealing the potential pathogenesis will help provide new treatment options for NSCLC. Previous studies have shown that lncRNAs participate in the pathogenesis of NSCLC.^27^, ^28^ In our study, we found a new lncRNA (lncRNA ENST00000558010.1, simplify to Lnc1) through comparison of the NSCLC tissues and paracancerous tissues in patients, and further revealed the function of Lnc1 in tumor progression. From our study, we found that the expression level of Lnc1 was positively correlated with the pathology of NSCLC. Lnc1 is highly expressed in NSCLC, especially in lung squamous cell carcinoma and adenocarcinoma. In addition, downregulation of Lnc1 inhibits the proliferation, migration and invasion abilities of NSCLC cells. By reviewing the relevant literature, we found that PLCB1 is significantly important in the regulation of various tumor cells.^29^, ^30^, ^31^ For example, Liang et al. found that PLCB1 promotes cholangiocarcinoma progression through the PLCB1‐PI3K‐AKT signaling axis.^32^ Wang et al. found PLCB1 enhances cell migration and invasion in gastric cancer via regulation of the actin cytoskeletal remodeling and epithelial‐mesenchymal transition.^33^ In our study, we confirmed that PLCB1 could promote migration and invasion in A549 and H1299 cells, and PLCB1 as an oncogene plays a role in the occurrence and development of NSCLC. In addition, PLCB1 is related to tumor differentiation and metastasis by analyzing the expression level of PLCB1 in clinical specimens. In addition, downregulation of PLCB1 could reverse the enhanced NSCLC cells proliferation, migration and invasion caused by the overexpression of Lnc1.

The Rap1 signal pathway has been reported to be correlated with the proliferation and adhesion of a variety of tumor cells,^34^, ^35^ such as colorectal cancer^24^ and NSCLC.^36^ Therefore, it is important to explore the Lnc1 expression and Rap1 signaling pathway activation in NSCLC. Our results showed that up regulation of Lnc1 expression activation the expression of Rap1 signal pathway marker proteins (rap1, c‐raf, erk1/2), while downregulation of PLCB1 can reverse the outcome. In addition, we used ERK‐IN‐3, which an inhibitor of Rap1 signaling pathway, can reverse the changes in cadherin caused by overexpression of Lnc1. In conclusion, our study determined that Lnc1 mediates the metastasis of NSCLC through regulating the PLCB1/Rap1 signaling pathway. Thus, Lnc1 plays an oncogenic role in the development of NSCLC and providing a new theoretical target for NSCLC. In addition, we performed a comprehensive investigation on an inhibitor of erk1/2. However, there are still some insufficiencies in the current research. First, the detailed mechanism of Lnc1 regulating EMT related proteins has not been explored. Second, we did not construct luciferase vector to dynamically observe Lnc1 and downstream molecule effect on tumor metastasis. Third, the expression level of Lnc1 in small cell lung carcinomas (SCLC) was not examined. Key experiments should continue to be performed in the future, such as Lnc1 regulating EMT, lncRNA interaction. In addition, Lnc1 as a target to therapy patient‐derived xenografts of NSCLC should be performed.

In conclusion, the present study showed that the expression level of Lnc1 is increased in NSCLC tissues compared to paracancerous tissues. Functional analyses further showed that Lnc1 mediates the metastasis of NSCLC through regulating the PLCB1/Rap1 signaling pathway. The detailed mechanism of Lnc1 regulating EMT related proteins will be investigated in our further research. In short, our result identifies current understanding of progression in NSCLC at the RNA level.

## AUTHOR CONTRIBUTIONS

Shenhui Dai designed the study and performed the experiments. Zhipeng Chen, Qiao Wang and Yin Lyu analyzed the data. Hao Zhang reviewed and edited the manuscript. All authors read and approved the final manuscript.

## FUNDING INFORMATION

The study was supported by the Social Development Projects of Key R&D Programs in Xuzhou City (KC22097).

## CONFLICT OF INTEREST STATEMENT

The authors declare that the research was conducted in the absence of any commercial or financial relationships that could be construed as a potential conflict of interest.

## Supporting information


**Figure S1.** (a) CCK‐8 assay was performed to evaluate the effect of ADCY7 on the proliferation of A549 cells. (b) Transwell assay was used to measure the effect of ADCY7 on migration ability on A549 cells. (c) Lnc1 pulldown nuclear proteins of A549 cells were separated by Coomassie blue staining. (d) The relative expression of Lnc1 knockdown and overexpression have been identified by RT‐qPCR analysis. (e) The statistical results of lung metastatic nodes in shLnc1 and shControl groups. (f, g) The data analysis of wound healing assay.

## Data Availability

All datasets generated for this study are included in the article/supplementary material. The datasets used and/or analyzed during the current study are available from the corresponding author on reasonable request.
