# Supplementary material for: LncRNA AC100826.1 regulated PLCB1 to promote progression in non‐small cell lung cancer
Source: Thorac Cancer. 2024 May 22;15(19):1477–89. doi: 10.1111/1759-7714.15323 (PMC11219295; doi:10.1111/1759-7714.15323)
Supplement: Supplementary file 1 — Figure S1. (a) CCK‐8 assay was performed to evaluate the effect of ADCY7 on the proliferation of A549 cells. (b) Transwell assay was used to measure the effect of ADCY7 on migration ability on A549 cells. (c) Lnc1 pulldown nuclear proteins of A549 cells were separated by Coomassie blue staining. (d) The relative expression of Lnc1 knockdown and overexpression have been identified by RT‐qPCR analysis. (e) The statistical results of lung metastatic nodes in shLnc1 and shControl groups. (f, g) The data analysis of wound healing assay. [file TCA-15-1477-s001.docx]

**LncRNA AC100826.1 regulated PLCB1 to promote progression in non-small cell lung cancer**

Shenhui Dai^1,2^, Qiao Wang^1,2^, Yin Lv^1,2^, Zhipeng Chen^2^, Xiucheng Liu^1,2^, Guoqing Zhao^1,2^, Hao Zhang^1,2*^

1. Thoracic Surgery Laboratory, Xuzhou Medical University, Xuzhou 221006, Jiangsu Province, China;

2. Department of Thoracic Surgery, Affiliated Hospital of Xuzhou Medical University, Xuzhou 221006, Jiangsu Province, China.

*Corresponding authors. Hao Zhang, Department of Thoracic Surgery, Affiliated Hospital of Xuzhou Medical University, 99 West Huaihai Road, Xuzhou 221006, Jiangsu Province, China. Tel: +8617798835177; Email: zhanghao@xzhmu.edu.cn.

**Figure (A)** CCK-8 assay was performed to evaluate the effect of ADCY7 on the proliferation of A549 cells. **(B)** Transwell assay was used to measure the effect of ADCY7 on migration ability on A549 cells. **(C)** Lnc1 pull-down nuclear proteins of A549 cells were separated by Coomassie Blue Staining. **(D)** The relative expression of Lnc1 knockdown and overexpression have been identified by RT-qPCR analysis. **(E)** The statistical results of lung metastatic nodes in shLnc1 and shControl groups. **(F,G)** The data analysis of wound healing assay.
